# Trends of Coagulation Parameters in Human Immunodeficiency Virus Patients

**DOI:** 10.3390/medicina59101826

**Published:** 2023-10-13

**Authors:** Bashir Abdrhman Bashir, Mohamed Hassan Mohamed, Mohamed A. Hussain, Wadah Osman, Ramzi A. Mothana, Sidgi Hasson

**Affiliations:** 1 Department of Hematology, Faculty of Medical Laboratory Sciences, Port Sudan Ahlia College, Port Sudan 33312, Sudan; 2 Department of Hematology, Faculty of Medical Laboratory Science, National University, Khartoum 11111, Sudan; mhassan0210@gmail.com; 3 Department of Pharmaceutical Microbiology, Faculty of Pharmacy, International University of Africa, Khartoum 11111, Sudan; mkasamber@gmail.com; 4 Department of Pharmacognosy, Faculty of Pharmacy, University of Khartoum, Al-Qasr Ave., Khartoum 11111, Sudan; w.osman@psau.edu.sa; 5 Department of Pharmacognosy, College of Pharmacy, King Saud University, Riyadh 11451, Saudi Arabia; rmothana@ksu.edu.sa; 6 School of Pharmacy and Biomolecular Sciences, Liverpool John Moores University, Liverpool L3 3AF, UK; s.s.hasson@ljmu.ac.uk

**Keywords:** human immunodeficiency virus (HIV), TTP, antiphospholipid, coagulation tests, Sudan

## Abstract

*Background and Objectives:* HIV disease is recognized to cause inconsistencies in coagulation via various pathways during infection. Some studies have indicated that HIV-infected patients are prone to developing thrombocytopenia, thrombosis, or autoantibodies that may cause difficulties in diagnosis. This study is intended to measure the trend of coagulation parameters in Sudanese patients with HIV. *Materials and Methods:* A cross-sectional study was carried out in patients with HIV admitted to the Sudan National AIDS Program (SNAP) from January 2018 to December 2019. Prothrombin time (PT), partial thromboplastin time (PTT), thrombin time (TT), D-dimer (DD), hemoglobin (HB), total lymphocyte count (TLC), platelet count (PLT), and a disintegrin and metalloproteinase with thrombospondin type 1 motif, member 13 (ADAMTS13), were evaluated among HIV Sudanese patients. *Results:* Out of the 44 HIV patients included, 6 (13.6%) were found to have thrombotic thrombocytopenic purpura-like events and 12 (27.2%) had antiphospholipid antibodies, of whom 8 (66.6%) showed anticardiolipin antibody (1gG (75%) and IgM (25%)) and 4 showed lupus anticoagulants. The HB, TLC, and PLT values were found to be significantly lower in HIV patients than in control (*p* = 0.000, 0.000, and 0.050, respectively). The PT and ADAMTS13 values showed no significant difference between HIV patients and control (*p* = 0.613 and 0.266, respectively). The PTT, TT, and DD values were found to be augmented in HIV patients versus the control (*p* = 0.000). *Conclusions:* Thrombotic thrombocytopenic purpura-like events among HIV Sudanese patients were explored. In addition, antiphospholipid antibodies were strikingly seen in these patients. Additional research is anticipated to confirm these diagnoses.

## 1. Introduction

The most widely recognized difficulty of human immunodeficiency (HIV) infection is blood clotting irregularities. As the disease advances, these anomalies become more articulated [1]. Thrombotic thrombocytopenic purpura (TTP) is brought on by a decline in or absence of the enzyme a disintegrin and metalloproteinase with thrombospondin type 1 motif, member 13 (ADAMTS13) activity [1]. TTP can be either innate or acquired. Acquired TTP is more prevalent than the congenital sort and is brought about via autoantibodies targeting ADAMTS13. HIV, antiplatelet drugs, immunosuppressive agents, and pregnancy are the most often recorded promoters of ADAMTS13 autoantibody arrangement causing acquired TTP [2]. TTP is an intriguing, hazardous type of microangiopathic hemolysis that can be related to HIV infection and has recently been evidenced to be related to low CD4 counts. The major immunological complication of HIV infection is cell depletion of CD4+ T lymphocytes, in which different mechanisms of causality have been suggested, including HIV-induced cytolysis, cytokine deregulation, T lymphocyte cytotoxic responses, and HIV-induced autoimmune responses [2]. Recently, Omoregie et al. found that HIV-infected patients with CD4 counts below 200 cells/µL had higher prothrombin time (PT) and partial thromboplastin time (PTT) values, though only PT corresponded to CD4 counts [3]. They thought that the advancement of HIV infection, therefore, leads to endothelial dysfunction and liver damage that could contribute to excessive clotting [3]. Another study also revealed that 34.9% of the blood sampled from HIV-infected individuals had deranged PTT. Nine in ten individuals with abnormal PTT also experience thrombotic disorders because of the presence of circulating anticoagulants [4]. Still, another study by Dikshit et al. found no clotting abnormalities in HIV patients, indicating the demand for further studies to clarify the impact of HIV infection as well as the state of coagulation profile of these patients [5]. The medical literature indicates that HIV-infected patients are predisposed to develop thrombotic thrombocytopenic purpura (TTP) and antiphospholipid antibodies (aPL) [6,7]. HIV presents an expanded hazard for acquiring TTP, with a 15–40-fold higher occurrence in HIV patients than the HIV-uninfected population; however, the pathogenesis is ineffectively comprehended [7]. These important subtypes mentioned above can be identified via immunoassay and useful coagulation tests for lupus anticoagulant (LA), anticardiolipin antibodies (aCL), and anti-beta 2 glycoprotein antibodies (anti-β2GPI) [6]. Becker et al. provided details regarding the commonness of aPL in HIV contamination. aCL was stated to be present in 0–94% of HIV patients, anti-β2-GPI in 4–47%, anti-prothrombin (aPT) in 2–12%, and LA in 0–53.5%. Very limited data exist on the spread of aPL in African patients with HIV [8]. It was suggested by Oudenhoven et al. that the total number of lymphocytes can be used as an alternate clinical marker rather than CD4 count. It gives insight during the evaluation of the clinical progression of the disease and response to treatment, as well as being employed in resource-limited settings [9]. This study was undertaken to measure the trends of certain coagulation parameters among Sudanese patients with HIV.

## 2. Material and Methods

A cross-sectional survey was conducted in the Sudan National AIDS Program (SNAP), Red Sea State, Sudan during the period from January 2018 to December 2019. Only 44 HIV-infected patients admitted to SNAP, not on antiretroviral therapy, who conformed to the study were selected, along with 31 healthy HIV-negative volunteers without any conspicuous symptoms. They were subjected to investigations of the values for prothrombin time (PT), partial thromboplastin time (PTT), thrombin time (TT), D-dimer (DD), hemoglobin (HB), platelet count (PLT), total lymphocyte count (TLC), lactate dehydrogenase (LDH), creatinine, and peripheral blood smear, as well as measurement of a disintegrin and metalloproteinase with a thrombospondin type 1 motif, member 13 (ADAMTS 13). HIV-seropositive subjects were already diagnosed through enzyme-linked immunosorbent assay and Western blot HIV assay. Unfortunately, CD4/CD8 count and HIV viral load were not measured due to shortage of facilities. Ethical approval was obtained from the State Ministry of Health and SNAP, Sudan. Written informed consent was obtained from all participants of the study. The reference values were PT 11–16 s, PTT = 25–43 s, TT = 15–22 s, DD = 0.1–0.3 mg/L, HB = 11.5–16.1 g/dL, TLC = 1.000–3.500 cells/µL, PLT = 150–400 cells/µL, LDH = 240–450 u/L, ADAMTS13 = 0.4–1.3, and creatinine = 0.5–1.6 mg/dL.

### 2.1. Inclusion and Exclusion Criteria

**Figure 1:**
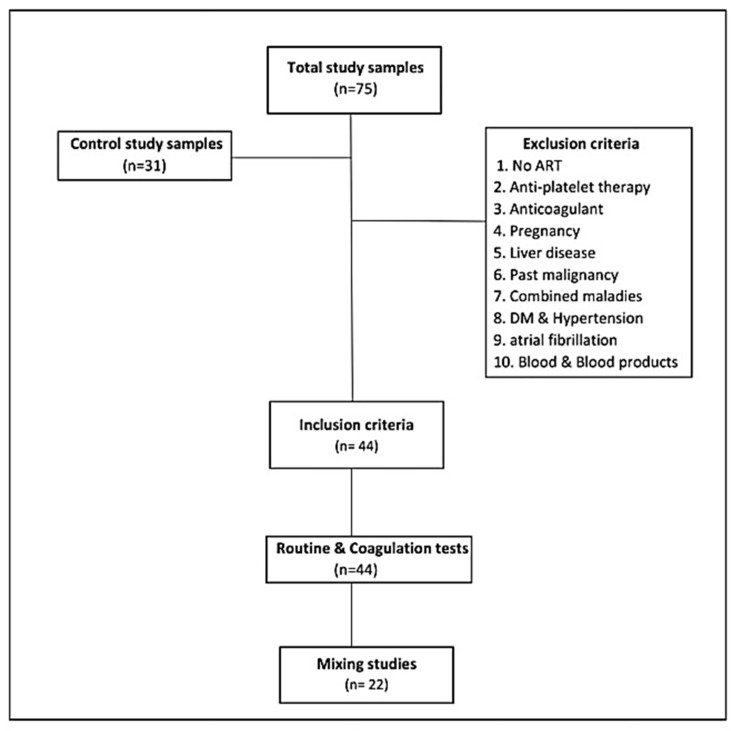
Flowchart of the work methodology.

This study included immunologically newly confirmed HIV-infected adults who had not initiated antiretroviral treatment, because antiretroviral drugs affect coagulation tests [10]. All subjects who were not willing to participate in the study, pregnant women, patients on anticoagulant or antiplatelet therapy, patients who had liver diseases (liver sickness impacts coagulation elements synthesis and diminishes the manufacture of vitamin K), patients with any existing or past malignancy or combined maladies, including hypertension, diabetes mellitus, and atrial fibrillation (all the previous are prothrombotic diseases), and patients receiving blood and blood product transfusion or antiretroviral therapy (ART) administrators were excluded from the study (Figure 1).

### 2.2. Laboratory Methods

First, 6 ml venous blood samples were aseptically gathered from each patient, out of which 3 mL was put in di-potassium ethylene diamine tetra acetic acid (K_2_EDTA) for estimated HB, TLC, and PLT and the remaining 3ml was treated with 3.2% tri-sodium citrate vacuum tube in a proportion of 1:9, following which it was immediately mixed via gentle reverse uniform inversion and centrifuged at room temperature at 3500 revolutions per minute for 15 min to obtain platelet-poor plasma. Then, we proceeded with the performance evaluation of PT, PTT, TT, and DD. The coagulation tests including PT, PTT, and TT were performed using a URIT coagulation analyzer, while the DD test was measured quantitatively via NycoCard^®^ reagent through NycoCard^®^ READER II (SN 67498, Axis-Shield PoC AS, Oslo, Norway). Monoclonal anti-GST with specific antibodies via enzyme-linked immunosorbent assays (ELISA test using the sensitive TECHNOZYM^®^ ADAMTS 13 Activity kit (Technoclone GmbH, Vienna, Austria)) was used to measure ADAMTS 13. HIV- infected subjects or HIV-negative controls with persistently prolonged PT, PTT, or TT values following the mixing correction test were additionally examined for antiphospholipid antibodies, specifically anticardiolipin antibodies and lupus anticoagulant (isotypes IgM and IgG), assayed using an ELISA kit (Orgentec Diagnostika, Mainz, Germany) using a GEA microplate ELISA reader, Barcelona, Spain.

### 2.3. Quality Control

Standard operating procedures (SOPs) and manufacturer directions were carefully followed throughout. The methods and all reagents were stored and arranged according to the manufacturer guidelines. The laboratory quality of every test was checked by running control materials before using the HIV samples. The examination of patients was performed when the strategies passed the quality control check.

### 2.4. Data Analysis

The data were entered into a statistical package for social sciences software (SPSS 24 version, IBN, Chicago, IL, USA) for analysis. The Kolmogorov–Smirnov normality test was applied to see the distribution of continuous variables and it was found that the variables were not normally distributed in each group. Nonparametric tests, a Mann–Whitney test, and a Wilcoxon signed–rank test was applied for the comparison of coagulation parameters between groups. Spearman’s rank-order correlation analysis was employed to assess the correlation of D-dimer and ADAMTS 13 with the studied parameters. The findings were tabulated as a mean ± standard deviation (SD). Receiver operating characteristic (ROC) curves were developed to determine sensitivity and specificity based on the area below the curve (AUC) and 95% confidence intervals. Accordingly, the cutoff points of PTT and D-dimer were 33.6 s and 0.1 mg/L, respectively. Tables are used to show the summarized information. A *p* value less than 0.05 was accepted as statistically significant for this study.

## 3. Results

A total of 44 HIV-infected subjects and 31 non-HIV subjects as controls were recruited. The HIV-infected subjects were aged between 18 and 65 years with a mean age of 33 ± 11.2 years; of them, 30 (68.2%) were males and 14 (31.8%) were females. The control subjects were also aged between 19 and 76 years with a mean age of 24.5 ± 10.3 years, of which 26 (83.9%) were males and 5 (16.1%) were females.

Routine laboratory investigations

Although within the normal range, the mean values of the HB, TLC, and PLT of the HIV-infected patients were significantly reduced compared to the controls (*p* = 0.000, 0.000, and 0.049, respectively). Interestingly, the TLC values were strongly correlated with the PTT, TT, and DD values (*r* = −0.436; *p* < 0.000, *r* = −0.480; *p* < 0.000, *r* = −0.416; *p* < 0.000, respectively). The mean values of LDH and creatinine yielded no significant difference between HIV-infected patients and controls (*p* = 0.078, 0.481, respectively) (Table 1). Only 6 (13.6%) of HIV-infected patients had laboratory evidence of renal dysfunction, and their median creatinine value was 0.89 mg/dL (range 0.44–2.9 mg/dL). Seven (15.9%) of the anemic HIV-infected patients were significantly different from the control, where their values of LDH were found to be elevated (*p* = 0.000). Among HIV-anemic patients, six of seven (85.7%) presented laboratory evidence of microangiopathic hemolytic anemia with remarkable schistocytosis (erythrocyte fragments) on the peripheral blood smear.

The mean PT values were 13.5 ± 2.1 s (range 9.7–22.8 s) (Table 1). The prothrombin clotting time values were short in 2 (4.5%), prolonged in 2 (4.5%), and normal in 40 (91%) of the HIV-infected patients (Table 2). Meanwhile, the PTT values were significantly higher in HIV-infected patients (*p* = 0.000) (Table 1): the mean PTT values were 41.7 ± 12.1 s (range 22.7–80.1 s) (Table 1). The partial thromboplastin clotting time values were prolonged in 14 (31.8%), short in 3 (6.8%), and normal in 27 (61.4%) of the HIV-infected patients (Table 2). The PTT and PT values were prolonged together in 2 (4.5%) of the HIV-infected patients. The PTT and TT values were also prolonged together in 3 (13.6%) of the HIV-infected patients. Meanwhile, the TT values were significantly increased in HIV-infected patients (*p* = 0.000) (Table 1): the mean TT values were 19.4 ± 4.8 s (range 15.1–41.0 s). The thrombin clotting time values were prolonged in 6 (13.6%) and normal in 38 (86.4%) of the HIV-infected patients (Table 2).

**Figure 2:**
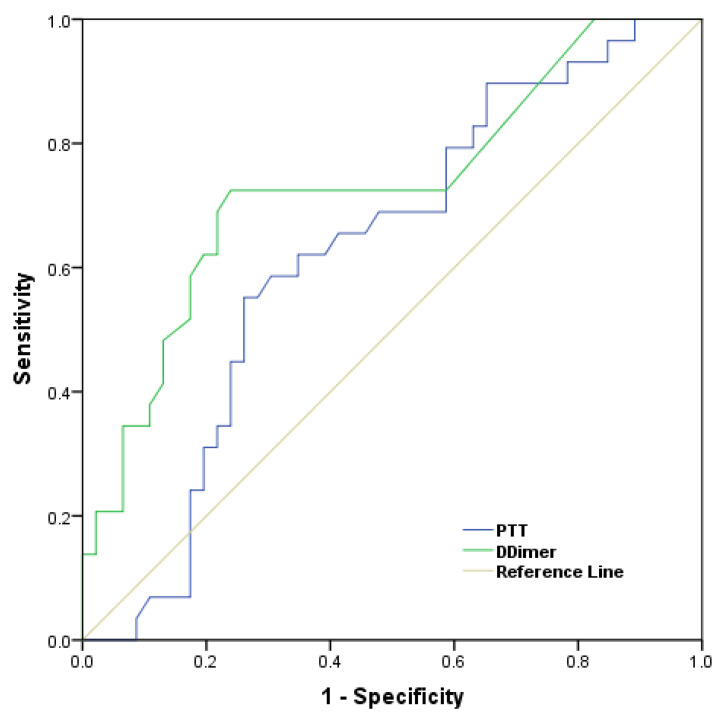
ROC curve analysis of plasma PTT and D-dimer cutoff optimization.

An ROC curve was generated to assess the clinical diagnostic accuracy for PTT and DD in the HIV-infected patients. For PTT and DD, the ROC curves explored (AUC: 0.624, sensitivity: 66%, specificity: 46%, cut-off: 33.6 s, 95% CI, 0.495–0.753) and (AUC: 0.731, sensitivity: 100%, specificity: 83%, cut-off: 0.1 mg/L; 95% CI: 0.610–0.853), respectively (Figure 2).

Mixing coagulation studies

Out of the 22 HIV-infected patients with prolonged hemostatic outcomes, 16 (72.7%) patients were rechecked to verify the causes of the extreme prolongation of PT and PTT tests using mixing studies. In our interpretations of the mixing study data, step 1 (using pooled normal plasma) and step 2 (using aged and absorbed plasma) of the findings highlighted no correction in 12 patients, which suggests the presence of antibodies that interfere with the coagulation tests (phospholipid inhibitors). 

Specific coagulation tests

The mean values of DD were higher in HIV-infected patients versus the controls (*p* = 0.000) (Table 1). We found that 31 (70.5%) of HIV-infected patients had elevated values of DD. The DD values were positively correlated with PTT and TT values (*r* = −0.378; *p* < 0.011, *r* = 0.353; *p* < 0.019, respectively) and negatively correlated with PLT, PT, and ADAMTS 13 (r = 0.108; *p* < 0.486, *r* = −0.208; *p* < 0.176, and *r* = 0.019; *p* < 0.904, respectively). Meanwhile, the ADAMTS 13 values were lower in the HIV-infected patients in comparison to control (*p* = 0.284) (Table 1). Fourteen (31.8%) of the HIV-infected patients had decreased levels of ADAMTS 13. The ADAMTS 13 values were positively correlated with PLT values (*r* = 0.528; *p* < 0.000), HB values (*r* = 0.476; *p* < 0.001), LDH values (*r* = −0.0336; *p* < 0.026), and creatinine levels (*r* = −0.323; *p* < 0.033). More minutiae of the specific coagulation variables are stated in Table 2.

TTP and Antiphospholipid syndrome

**Figure 3:**
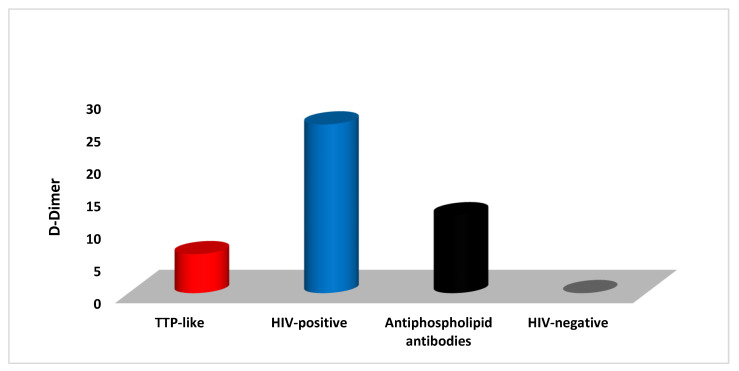
High D-dimer levels noted in TTP-like (*n* = 6), antiphospholipid antibodies (*n* = 12), and other HIV-positive (*n* = 26) patients. HIV-negative (control) had a normal D-dimer level.

Six (13.6%) out of the forty-four HIV-infected patients had simultaneous anemia, schistocytosis in blood smear, thrombocytopenia, high DD, dropping in ADAMTS 13, and high levels of LDH (a marker of hemolysis). These outcomes suggest the presence of acquired TTP among the underlying HIV disease. In this study, most of the TTP-like cases were seen in females (66.6%). All TTP-like cases were associated with increased levels of DD. Twelve (27.2%) of the forty-four HIV-infected patients were positive for antiphospholipid (aPL). Eight patients had aPL presented anticardiolipin antibodies with a mean IgG cutoff (of 18.0 ± 1.2 U/mL) and a mean IgM cutoff (of 26.3 ± 2.3 U/mL). The anticardiolipin IgG isotype was detected in six (75%) of the HIV patients and the IgM isotype in two (25%) of the HIV patients. Only four patients with lupus anticoagulant were detected in all cases. Of the 12 aPL patients, 4 were males and 8 were females. The D-dimer concentrations in the associated underlying conditions are explored in Figure 3.

Ultimately, the potential ailments that present in HIV-infected patients in this study are emphasized in Table 3.

## 4. Discussion

Hematological alterations are prominent complications of HIV disease and impact the blood cyte lineages, causing anemia and/or leukopenia and/or thrombocytopenia [11]. Furthermore, changes in coagulation mechanisms have been declared in HIV cases.

In this study, the PLT values were significantly lower in HIV-infected patients compared to the control (HIV-negative). This finding is in concordance with studies by Okoroiwu et al. and Obeague et al. [12,13]. The inhibition of thrombopoiesis, generation of the immune complex, and presence of antiplatelet antibodies (resulting in the increased rate of platelet destruction in the blood circulation) is thought to be the likely mechanism underlying thrombocytopenia in HIV infection. The invasion of megakaryocytes by HIV (owing to the presence of HIV receptors) causes impaired thrombopoiesis [10]. In regions where viral loads and CD4 counts are missing, utilizing the ongoing WHO rules that propose the utilization of TLC in conjunction with clinical data as a model is the next choice. Total lymphocyte count values were also exhibited by our study, reduced in HIV-infected patients versus the control. However, our findings are consistent with a previous study [9]. This study shows that TLC can offer insightful information for several reasons. In cases where CD4 testing may be limited or unavailable, this makes TLC a more accessible marker (easy and affordable). The second is cost-effectiveness; although CD4 requires specialized equipment and trained workers, TLC reduces expenses and more effectively uses resources. Third, TLC can be used to monitor the development of the disease. A reduction in TLC may signal immune system deterioration and point to the need for additional assessment and treatment. Fourth, TLC can be used to screen for opportunistic infections; TLC can be used to spot people who could need preventative care or more investigation for potential infections. Fifth, TLC is a straightforward measurement with a simple interpretation, thus it is simple and easy to use. It can be easily understood by healthcare providers. However, we point out that while CD4 count is still the gold standard for evaluating immune function in HIV/AIDS patients, utilizing TLC as a substitute clinical measure in a constrained situation can offer insightful information for disease monitoring and response assessment. Anemia, thrombocytopenia, microvascular thrombosis, and/or various organ dysfunctions are the hallmarks of microangiopathic hemolytic anemia. Usually, it happens when the HIV condition is further advanced. This study reported that six (13.6%) patients had this condition, comparable to the discovery made by Dineshkumar et al. in their case [14]. According to a study performed in Milan [5], the incidence of microangiopathic hemolytic anemia was 1.4% before the introduction of ART; however, there were no cases reported throughout the ART era. Serious consequences of microangiopathic hemolytic anemia correlated with HIV infection might range from functional and quality-of-life impairments to links to disease progression and shortened survival [15].

Renal dysfunction is a frequent consequence in HIV patients and can be brought on by several conditions, such as immunological complex kidney disorders, drug nephrotoxicity, and HIV-associated nephropathy (HIVAN). The risk of kidney damage in patients with HIV is further increased by uncontrolled HIV infection and coinfection with the hepatitis C virus (HCV) [16]. It was reported that HIV-infected patients receiving antiretroviral medication frequently experienced renal impairment [17]. Of note, our study reveals that only six (13.6%) HIV-infected patients had evident laboratory remarks of kidney dysfunction and no HIV-infected patients had obvious clues of neurological dysfunction. Information on renal dysfunction in HIV patients not receiving antiretroviral therapy (ART) is, however, scarce.

HIV infection frequently results in coagulation abnormalities; thus, we eliminated any patients who had started taking antiretroviral therapy (ART). ART improves HIV mortality but exacerbated coagulopathies [10]. On the other hand, this study reveals that PT values were slightly higher in HIV-infected patients than the values obtained in controls. These results are similar to Omoregie et al. [3], although this was insignificant in our study. This insignificance may have resulted from the limited number of patients. Moreover, PTT values were demonstrated to be significantly higher in HIV-infected patients versus controls. Many previous studies also reported that PTT levels were prolonged [12,18]. There is often endothelial damage contributing to the activation and consumption of the blood clotting factors in HIV disease. Also, HIV disease may cause hepatic disruption, immune dysregulation, and the presence of antiphospholipid antibodies (LA and aCL). These anomalies may represent the prolongation of PTT values and also PT showed in HIV-positive subjects [18]. TT values were also found to be higher in HIV-infected patients than in the controls of our study. This finding strongly conforms to Ifeanyichukwu et al. [18]. This may be considered as a result of the presence of D-dimer at a high rate or instead influenced by other parameter(s) or other confounding factors. This study uncovered that DD levels are fundamentally higher in HIV patients when compared to control. These outcomes are identical to previous studies that pointed to the considerable increase in DD in HIV cases [19,20]. A high DD level suggests that the body has recently experienced clot formation and disintegration. Although hyperfibrinolysis and hypercoagulability can both be indicated by high DD levels, the test does not explicitly discriminate between the two [21]. On the other hand, ADAMTS 13 values in this study were found to be lower in HIV-infected patients versus the controls. These results were also found in early studies, suggesting that HIV disease has an inhibitory reaction to ADAMTS 13 [22,23].

Throughout the study, we diagnosed six cases of HIV that had TTP-like presentation. This finding was also detected early by Meirairy et al., Louw et al., and Dabson et al. [1,22,23]. The diagnosis of this TTP-like presentation is based on firstly, exclusion of the other microangiopathies such as disseminated intravascular coagulation (DIC) and hemolytic uremic syndrome (HUS). Secondly, it is based on the presence of thrombocytopenia, increased LDH, schistocytosis in the peripheral blood smear, decreased ADAMTS 13 values, and elevated DD values. DIC is not thought of when the coagulation test(s) is/are normal even in the presence of high DD values [24]. HUS would only be diagnosed in patients with present or historical diarrhea and extreme kidney dysfunction [25].

This study affirmed our clinical impression that high DD values without irregularities in coagulation parameters seem to be asymmetrical features of cases with HIV-related TTP. So, in our practice, this outcome is frequently useful in proposing the diagnosis. It is conceived that the extremely high DD values noted in HIV-related TTP reflect the contrast in the pathogenesis [6]. TTP associated with HIV-infected patients has some assumptions regarding the initial onset. Inflammatory cytokines such as tumor necrosis factors (α, β) and interleukins (6, 7) thoroughly trigger the endothelial cell to downregulate the release of ADAMTS 13. Likewise, the ADAMTS 13 that cleaves the ultra-large von Willebrand factor (ULVWF) is altered. This may eventually contribute to the inadequacy of ADAMTS 13 and the overexpression of ULVWF, causing the commencement of TTP [23]. In addition, HIV disease is associated with prevalent deficiencies in micronutrition that may cause diminished ADAMTS output [6]. Autoantibodies to ADAMTS 13 are additionally present in HIV-infected patients because of an impaired immune system. An inhibitory antibody may likewise cause the underlying onset of HIV-related TTP [26].

HIV disease is considered one of the commonest viral infections that induces the development of antiphospholipid antibodies. Antiphospholipids stimulated by the infection have been declared as being occasional (non-pathogenic) [8]. Most aPLs among HIV-infected patients have been reported as being transient outcomes and case reports [27]. Accidentally, our findings explored 12 aPL cases among HIV Sudanese patients. These findings resemble that of Palomo et al., who reported that anticardiolipin antibodies were present in 11.1% and negative lupus anticoagulant when studying both (anticardiolipin antibodies and lupus anticoagulant) [28]. Among the two subtypes of antiphospholipid antibodies performed in this study, anticardiolipin antibodies were seen as predominant, and only four cases with lupus anticoagulant were detected. The IgM antibody isotype was increased in the HIV-infected patients in the present study and these findings are dissimilar to those of Palomo et al. and Wincup et al. [28,29]. They reported an increased mean value of IgG antibodies in adult HIV patients. However, this may be regarded as an epiphenomenon of vaccination, infections, or the nutritional status of populations [30]. Our HIV-infected patients associated with these antibodies were not accompanied by an episode of thrombosis. The presence of aPL may predict the risk of developing thrombosis or complications of cardiovascular disease.

There are some constraints to this study, such as the small number of HIV patients included and the disregard for clinical indicators like CD4/CD8 counts and non-specific noteworthy markers for TTP-like presentation. In addition, experiments concerning von Willebrand factor values, tissue factor levels, and autoantibodies of the ADAMTS 13 ratio ought to be conducted in the future. 

## 5. Conclusions

In summary, TTP-like events among HIV Sudanese patients are suggested. Moreover, autoimmune phenomena such as antiphospholipid antibodies are also interestingly associated with HIV Sudanese patients. Further research is needed to establish these diagnoses.

## Figures and Tables

**Table 1:** Baseline data on HIV-infected versus HIV-negative group.

Variables	HIV Patients (*n* = 44)	HIV-Negative (*n* = 31)	*p*
**Age** (mean ± SD) (range)	33.0 ± 11.2 18–65	24.5 ± 10.3 19–76	0.000
**Sex** (male) (female)	30 (68.2%) 14 (31.8%)	26 (83.9%) 5 (16.1%)	0.126
**HB** level g/dL	11.2 ± 2.0	14.2 ± 0.9	0.000
**PLT** × 10^9^/L	225.8 ± 106.7	263.2 ± 64.3	0.049
**TLC** × 10^9^/L	1663 ± 860	5106 ± 1466	0.000
**PT** second	13.5 ± 2.1	13.1 ± 1.4	0.613
**PTT** second	41.7 ± 12.1	30.8 ± 4.3	0.000
**TT** second	19.4 ± 4.8	16.4 ± 2.3	0.000
**DD** mg/L	2.99 ± 5.2	0.13 ± 0.06	0.000
**Creatinine** mg/dL	1.04 ± 0.56	0.87 ± 0.28	0.481
**LDH** U/L	372.7 ± 148.2	321.7 ± 67.6	0.078
**ADAMTS** 13 iu/mL	0.52 ± 0.39	0.61 ± 0.31	0.284

HB: hemoglobin, PLT: platelet count, TLC: total lymphocyte count, PT: prothrombin time, PTT: partial thromboplastin time, TT: thrombin time, DD: D-dimer, LDH: lactate dehydrogenase, ADAMTS 13: a disintegrin and metalloproteinase with a thrombospondin type 1 motif, member 13.

**Table 2:** Summary of the outcome of coagulation studies.

Character/Parameters	PT (*n* = 44)	PTT (*n* = 44)	TT (*n* = 44)	DD (*n* = 44)	ADAMTS 13 (*n* = 44)
Short (low)	2 (4.5%)	3 (6.8%)	−	−	14 (31.8%)
Normal	40 (91%)	27 (61.4%)	38 (86.4%)	13 (29.5%)	30 (68.2%)
Prolong (high)	2 (4.5%)	14 (31.8%)	6 (13.6%)	31 (70.5%)	−
Median	13.4	41.7	19.4	0.90	0.49
Range of test	9.7–22.8	22.0–80.1	15.1–41.0	0.10–20.0	0.02–1.25
Range of control (*n* = 31)	10.6–16.2	22.0–39.0	12.9–22.2	0.03–0.30	0.10–1.10
Reference interval	11.0–16.0	25.0–43.0	15–22.0	0.1–0.3	0.4–1.3

PT: prothrombin time, PTT: partial thromboplastin time, TT: thrombin time, DD: D-dimer, LDH: lactate dehydrogenase, ADAMTS 13: a disintegrin and metalloproteinase with a thrombospondin type 1 motif, member 13.

**Table 3:** Potential outcomes for the HIV-infected patients in this study.

Conditions	HIV-Infected Patients (*n* = 44)
Renal dysfunction	6 (13.6%)
Microangiopathic hemolytic anemia	6 (13.6%)
Hypercoagulability + hyperfibrinolysis state (**High D-dimer**)	31 (70.5%)
Coagulation factor deficiencies	10 (22.7%)
Coagulation factor inhibitors	12 (27.3%)
Thrombocytopenia	8 (18.2%)
TTP-like	6 (13.6%)
Antiphospholipid	12 (27.3%)
(Anticardiolipin)	8 (18.2%)
(Lupus anticoagulant)	4 (9.1%)

## Data Availability

Most of the relevant data are available in the main text; further data are available from the corresponding author upon reasonable request.
